# Inoculating Against Fake News About COVID-19

**DOI:** 10.3389/fpsyg.2020.566790

**Published:** 2020-10-23

**Authors:** Sander van der Linden, Jon Roozenbeek, Josh Compton

**Affiliations:** ^1^Cambridge Social Decision-Making Laboratory, Department of Psychology, School of Biology, University of Cambridge, Cambridge, United Kingdom; ^2^Institute for Writing and Rhetoric, Dartmouth College, Hanover, NH, United States

**Keywords:** COVID-19, fake news, misinformation, inoculation, infodemic

## Abstract

The outbreak of the SARS-CoV-2 novel coronavirus (COVID-19) has been accompanied by a large amount of misleading and false information about the virus, especially on social media. In this article, we explore the coronavirus “infodemic” and how behavioral scientists may seek to address this problem. We detail the scope of the problem and discuss the negative influence that COVID-19 misinformation can have on the widespread adoption of health protective behaviors in the population. In response, we explore how insights from the behavioral sciences can be leveraged to manage an effective societal response to curb the spread of misinformation about the virus. In particular, we discuss the theory of psychological inoculation (or *prebunking*) as an efficient vehicle for conferring large-scale psychological resistance against fake news.

## Introduction

The emergence of the novel coronavirus (SARS-CoV-2) in December of 2019 has quickly led to a global pandemic claiming hundreds of thousands of deaths worldwide already (Roser et al., 2020). In the absence of an effective treatment or vaccine, researchers have pointed out that managing the pandemic response will require leveraging insights from the social and behavioral sciences, particularly with regard to non-pharmaceutical interventions and containing the spread of misinformation about COVID-19 (Depoux et al., 2020; Habersaat et al., 2020; Van Bavel et al., 2020). In fact, the spread of misleading information about the virus has led the World Health Organization (WHO) to warn of an on-going “infodemic” or an overabundance of information—especially misinformation—during an epidemic (World Health Organization, 2020b; Zarocostas, 2020). This makes it harder for people to find trustworthy and reliable information when they need it. In this article, we ask three critical questions to help better inform societal response to the infodemic, namely; (1) what is the scope and reach of misinformation about COVID-19 in the general population, (2) what evidence is there to suggest that misinformation about the virus is undermining public support for—and the adoption of—preventative health behaviors; and (3) how can insights from psychology be leveraged to effectively manage societal response to help limit the spread of influential misinformation? In particular, in order to “immunize” people against the misinformation virus we draw on the theory of psychological inoculation and its real-world application.

## Misinformation About COVID-19

Misinformation about COVID-19 has proliferated widely on social media, ranging from the peddling of fake “cures,” such as gargling with lemon or salt water and injecting yourself with bleach (World Health Organization, 2020a), to false conspiracy theories that the virus was bioengineered in a lab in Wuhan (Andersen et al., 2020; Cohen, 2020), or that the 5G cellular network is causing or exacerbating symptoms of COVID-19 (BBC News, 2020). The conspiracy film “*Plandemic*” appeared online on May 4th of 2020, garnering millions of views and quickly becoming one of the most widespread examples of coronavirus-related misinformation (Cook et al., 2020). The video promotes dangerous health advice, for example, falsely suggesting that wearing a mask actually “activates” the coronavirus. Fake news about the virus has also been actively promoted by political elites, such as President Trump and Brazilian President Jair Bolsonaro, who falsely claimed that hydroxychloroquine is “working in all places” as a treatment against the virus (Constine, 2020). But misinformation about COVID-19 is not limited to information that is blatantly true or false, which widens the scope of the problem. For example, although the harms and benefits of hydroxychloroquine as a potential treatment are indeed being studied, there is currently no scientific consensus on its effectiveness (Geleris et al., 2020; Meyerowitz et al., 2020). Thus, even deciding what counts as misinformation about COVID-19 is a complicated matter, as insights into the causes of and treatments for the virus develop over time. Nonetheless, it is becoming increasingly clear that misinformation about COVID-19 is a common problem. For example, a poll by Ofcom in the United Kingdom found that almost half (46%) of the United Kingdom population reported exposure to fake news about the coronavirus (Ofcom, 2020). Similar results (48%) have been reported by Pew in the United States (Mitchell and Oliphant, 2020). In particular, amongst those exposed, nearly two-thirds (66%) reported seeing it on a daily basis, which is problematic as repeated exposure is known to increase belief in fake news (Pennycook et al., 2018). Although mass endorsement of conspiracy theories about the virus is not yet widespread, substantial minorities (typically about a third of the sample) in the United Kingdom and the United States report to believe that the virus is either manmade or produced on purpose by powerful organizations (Freeman et al., 2020; Roozenbeek et al., 2020b; Uscinski et al., 2020). Indeed, a YouGov survey found that about 28% of Americans and 50% of Fox News viewers think that Bill Gates is planning to use the COVID-19 vaccine to implement microchips in people (Sanders, 2020). Moreover, a recent analysis of the most viewed coronavirus YouTube videos found that over 25% of the top videos about the virus contained misleading information, reaching over 62 million views worldwide (Li et al., 2020).

## How is the Spread of Misinformation Harming Societal Response to the Pandemic?

Another emerging insight is that COVID-19 conspiracies and rampant misinformation can adversely impact the effectiveness of containment strategies. Indeed, misinformation about COVID-19 can fundamentally distort people’s risk perception of the virus (Krause et al., 2020). This is important as risk perception has been linked to the adoption of COVID-19 preventative health behaviors (Dryhurst et al., 2020). A recent study by Uscinski et al. (2020) found that belief in conspiracies about the virus is associated with a propensity to reject information from expert authorities. Similar findings were reported by Freeman et al. (2020), who also noted a link between belief in COVID-19 conspiracies and an increase in vaccine hesitancy. For example, people who endorsed the conspiracy that the virus is bioengineered were less likely to report compliance with public health guidelines (e.g., staying at home) and were less likely to report to accept a COVID-19 vaccine (see also Imhoff and Lamberty, 2020). These effects are problematic because at present polls show that only 50% of Americans are willing to get a potential vaccine if one becomes available, which undermines the potential for herd immunity against the coronavirus (Cornwall, 2020). Importantly, misinformation about the virus has been shown to have other serious societal consequences as well. Recent reports have indicated that coronavirus misinformation has been linked to mob attacks, mass poisonings (Depoux et al., 2020), and acts of vandalism (Spring, 2020). In the United Kingdom alone, people have set fire to least 50 phone masts in response to the 5G conspiracy (BBC News, 2020) and research finds that belief in the 5G conspiracy is linked to violent intentions (Jolley and Paterson, 2020). In addition, an analysis of over 60 million geo-coded cell phones found reduced social distancing in pro-government areas after Brazil’s president inaccurately portrayed the risks of COVID-19 (Ajzenman et al., 2020). Similar analyses have been conducted in the United States in response to political polarization over COVID-19 preventative health behaviors (Allcott et al., 2020), highlighting the disruptive potential of high-profile misinformation for both individual and societal well-being.

## Leveraging Insights From Psychology: Inoculating Against COVID-19 Misinformation

So far, little attention has been paid to insights from the social and behavioral sciences to combat misinformation about COVID-19, despite the ample availability of research to draw from Van Bavel et al. (2020). One insight that has emerged is that fact-checks tend to spread slower on social media than misinformation (Vosoughi et al., 2018), making it difficult for fact-checking to be effective on its own. A review by *Politico*, for example, found that Facebook’s fact-checking efforts did little to prevent coronavirus conspiracies from being shared widely in private groups on the platform (Scott, 2020). Further complications arise from the “continued influence effect” of misinformation, which states that people may continue to believe misinformation even after it has been debunked (Ecker et al., 2010; Lewandowsky et al., 2012). In addition, while media literacy initiatives are important and can be effective under the right conditions (Bode and Vraga, 2015; Guess et al., 2020; Van Bavel et al., 2020), they are often expensive to develop, slow to roll out, and *reactive* rather than *proactive*.

In particular, given the practical challenges of fact-checking and the difficulty of correcting misinformation after the damage is already done, researchers have started to explore *prebunking* (i.e., preemptive debunking). Because misinformation spreads through networks much like a real virus “infecting its host” and rapidly transmitting falsehoods from one mind to another, the natural antidote is a psychological vaccine against fake news (van der Linden and Roozenbeek, 2020).

### Inoculation Theory

The theory of psychological inoculation takes the historic practice of vaccination in medicine into the realm of resistance to persuasion (McGuire, 1964). In a medical inoculation, a virus is weakened to the point where it will not make the person sick, but it will trigger protective responses, like antibodies. In a persuasion inoculation, a strong challenge (e.g., a conspiracy theory) is weakened to the point where it will not change the person’s position—the person’s healthy state—but it will trigger protective responses, like enhanced critical thinking (McGuire, 1964; Compton, 2013). In both contexts, a similar process is at work: exposure to weakened challenges leads to resistance to stronger challenges. In psychological inoculation, the weakened challenge often consists of two elements (Compton, 2013), namely; (a) a forewarning of a threat or attack on one’s attitudes and (b) a preemptive refutation of counter-arguments (or prebunking). Preemptive refutation of misinformation weakens the misinformation, just as a medical vaccine is often comprised of weakened virus. For example, in a study on misinformation about climate change, participants were (a) forewarned that some political actors try to mislead people on the issue and (b) provided with facts and arguments to refute the misinformation—preemptively—that is, before they were exposed to a full dose of misinformation later on (van der Linden et al., 2017). The study found that the inoculation partially immunized people against climate misinformation (see also Maertens et al., 2020a).

A number of things happen during the inoculation process of resistance to influence. One of the most important is threat—the motivation to engage in resistance. In inoculation research, threat is a response to vulnerability (McGuire, 1964; Compton, 2013)—for example, when a preemptive inoculation message raises and refutes a persuasive attack (e.g., Banas and Richards, 2017), or when an inoculation message exposes reasoning fallacies (Cook et al., 2017). The cognitive and affective processes unleashed by threat are varied and powerful, including increased counterarguing (Pfau et al., 2006), increased attitude accessibility (Pfau et al., 2003), less psychological reactance against the inoculation-informed campaign (Richards and Banas, 2015), and more psychological reactance against attack messages (Miller et al., 2013). For conventional, *prophylactic* inoculation to take hold, the desired position needs to already be in place—a healthy state (Compton, 2013). This is the classic approach of inoculation theory. In the context of the coronavirus, this would imply protecting the attitudes of those people who are already following public health guidelines. Strengthening their attitudinal defenses will decrease the potency of misinformation attacks. However, a more recent approach within inoculation theory expands its efficacy to also include a *therapeutic* application—inoculation treatments that target an unhealthy state (Compton, 2020). New work in this latter area expands inoculation theory’s reach by inoculating audiences who have already been “afflicted” with the informational virus. Therapeutic inoculation works by boosting immune defenses and decreasing the probability that people will spread the virus. For example, people with skeptical attitudes toward climate science can still benefit from inoculation against misinformation in the sense that they generate stronger attitudes toward the scientific consensus (Cook et al., 2017; van der Linden et al., 2017).

The health domain boasts a particularly strong record for inoculation theory—appropriately enough in the context of COVID-19. Much of this work has looked at how inoculation theory-informed public health messages could help shore up resistance to unhealthy pressures, like smoking cigarettes (Pfau et al., 1992) or binge drinking (Parker et al., 2010). More recently, inoculation work has explored ways of enhancing beneficial health behaviors, like committing to exercise programs (Dimmock et al., 2016) or strengthening vaccination intentions (Wong and Harrison, 2014), especially in response to conspiracy theories (Jolley and Douglas, 2017). For example, vaccination intentions only improved when participants were presented with anti-conspiracy arguments *prior* to exposure to the vaccination conspiracy theories but not when presented with counter arguments afterward.

### Actively Inoculating Against Misinformation

**FIGURE 1:**
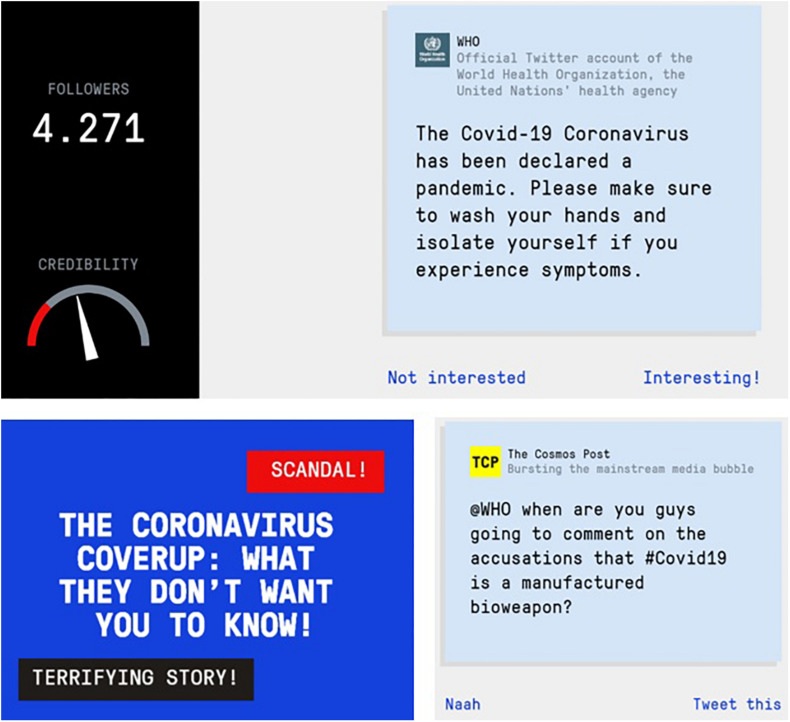
Screenshots from the *Bad News* game about coronavirus (www.getbadnews.com). Images and links reproduced with permission from *Bad News*.

Two further advances have been proposed in inoculation research that hold promise for the scalability and broad applicability of inoculation interventions, particularly in the context of misinformation: a renewed focus on active inoculations (McGuire and Papageorgis, 1961; Roozenbeek and van der Linden, 2018), and a shift in attention from inoculating against individual examples of unwanted persuasion (e.g., climate change or vaccination) to the manipulation *techniques* that underpin most fake news such as using emotional language (Brady et al., 2017), conspiratorial reasoning (Lewandowsky et al., 2013; van der Linden, 2015) or impersonating experts online (Goga et al., 2015). The idea behind active inoculation is to let people generate their own “antibodies.” A practical application of active inoculation theory is the award-winning online browser game *Bad News*.^1^ The game offers a simulated social media environment in which people take on the role of a fake news creator and learn about six common misinformation techniques over the course of six levels, or “badges” (for a detailed theoretical overview see Roozenbeek and van der Linden, 2019; van der Linden and Roozenbeek, 2020). The inoculation component in the game consists of a combination of (a) warnings about fake news and (b) pre-exposure to weakened doses of the techniques used in the production of fake news. Both processes can potentially increase the inoculation effect by facilitating retention in memory for longer periods of time (Pfau et al., 1997, 2005). Research has shown that *Bad News* significantly improves players’ ability to resist misinformation techniques after gameplay, and increase players’ confidence in spotting misleading information (Basol et al., 2020). In addition, in collaboration with the United Kingdom Foreign Office, the game has been translated internationally and its effectiveness as an inoculation intervention has been replicated across five different language versions (Roozenbeek et al., 2020c). The inoculation effect itself can last for months (Pfau and Bockern, 1994), including with regular “top-ups” or “booster shots” following gameplay (Maertens et al., 2020b). In response to the outbreak of the coronavirus pandemic, we altered the *Bad News* game’s “conspiracy” scenario to feature weakened doses of conspiracies about the virus. Figure 1 shows a number of screenshots from the game. Players are tasked with inventing and spreading a fake conspiracy theory about COVID-19, and learn about the negative consequences of their actions in the form of replies by social media users in their network, thus exposing how misinformation is created, spread and shared.

The relatively easy adaptation of the *Bad News* game to immunize people against misinformation specifically about the COVID-19 pandemic highlights the potential to translate theoretical laboratory findings into scalable real-world inoculation interventions: the game is played by about a million people worldwide (Roozenbeek et al., 2020c), thus “inoculating” a large number of people who voluntarily navigate to the *Bad News* website. Importantly, it is not necessary for every single individual to receive the “vaccine”: if enough people have developed antibodies against the techniques used to spread misinformation about COVID-19, in theory, societal herd immunity could be achieved.

## Conclusion

Prevention is better than cure. This is true as much for diseases as it is for the spread of misinformation. Although the *Bad News* game is a useful tool, more work is needed to curb the spread of misinformation about COVID-19, including a multi-layered defense system against “post-truth” science denial (van der Linden, 2019) which will include effective debunking and real-time rebuttal in addition to inoculation (Schmid and Betsch, 2019). A practical application of inoculation theory in the context of COVID-19 misinformation is the new online game, *Go Viral!*,^2^ developed in collaboration with the United Kingdom government and the WHO in which players learn to resist three manipulation techniques commonly used to spread misinformation about the coronavirus: fearmongering, the use of fake experts, and conspiracy theories. An open question in active inoculation research is the extent to which inoculation can boost *truth-discernment* skills, that is, not just the ability to spot and resist misinformation attacks but also the ability to better identify real or credible news (Guess et al., 2020; Roozenbeek et al., 2020a). Compton et al. (2016) called for more “work that pushes forward our understanding of persuasion and has applied value as a health messaging strategy to help combat serious threats to healthy living” (p. 1). Promoting accurate beliefs about COVID-19, and encouraging healthier, safer behaviors related to COVID-19 prevention, would certainly answer this call. Indeed, COVID-19 health messaging can harness both ways in which inoculation theory is used to protect healthier beliefs and actions: building resistance to unhealthy influence, like conspiracy theories, and encouraging healthier behaviors, like social distancing and wearing a mask in public. We look forward to future research on both prophylactic and therapeutic applications of psychological inoculation in the context of COVID-19.

## Data Availability Statement

The original contributions presented in the study are included in the article/supplementary material, further inquiries can be directed to the corresponding author.

## Author Contributions

SL and JR conceptualized the study and drafted the manuscript. JC co-authored the article and provided input on the final version of the manuscript. All authors contributed to the article and approved the submitted version.

## Conflict of Interest

The authors declare that the research was conducted in the absence of any commercial or financial relationships that could be construed as a potential conflict of interest.

## Footnotes

1 www.getbadnews.com

2 The game can be played for free at www.goviralgame.com.
